# Cell Aggregate Assembly through Microengineering for Functional Tissue Emergence

**DOI:** 10.3390/cells11091394

**Published:** 2022-04-20

**Authors:** Gozde Eke, Laurence Vaysse, Xi Yao, Mélanie Escudero, Audrey Carrière, Emmanuelle Trevisiol, Christophe Vieu, Christian Dani, Louis Casteilla, Laurent Malaquin

**Affiliations:** 1 Laboratoire d’Analyse et d’Architecture des Systèmes, Centre National de la Recherche Scientifique (LAAS-CNRS), Université de Toulouse, INSA, UPS, 31400 Toulouse, France; gekecevik@laas.fr (G.E.); emmanuelle.trevisiol@laas.fr (E.T.); cvieu@laas.fr (C.V.); 2 RESTORE Research Center, Université de Toulouse, INSERM 1301, CNRS 5070, EFS, ENVT, 31100 Toulouse, France; laurence.vaysse@inserm.fr (L.V.); melanie.escudero@inserm.fr (M.E.); audrey.carriere-pazat@inserm.fr (A.C.); louis.casteilla@inserm.fr (L.C.); 3 Institut de Biologie Valrose, Université Côte d’Azur, 06108 Nice, France; yao.xi@univ-cotedazur.fr (X.Y.); christian.dani@univ-cotedazur.fr (C.D.)

**Keywords:** cell spheroids, organoids, cell aggregates, functional microtissues, microfabrication, assembly technologies, micropatterning, microengineering technology

## Abstract

Compared to cell suspensions or monolayers, 3D cell aggregates provide cellular interactions organized in space and heterogeneity that better resume the real organization of native tissues. They represent powerful tools to narrow down the gap between in vitro and in vivo models, thanks to their self-evolving capabilities. Recent strategies have demonstrated their potential as building blocks to generate microtissues. Developing specific methodologies capable of organizing these cell aggregates into 3D architectures and environments has become essential to convert them into functional microtissues adapted for regenerative medicine or pharmaceutical screening purposes. Although the techniques for producing individual cell aggregates have been on the market for over a decade, the methodology for engineering functional tissues starting from them is still a young and quickly evolving field of research. In this review, we first present a panorama of emerging cell aggregates microfabrication and assembly technologies. We further discuss the perspectives opened in the establishment of functional tissues with a specific focus on controlled architecture and heterogeneity to favor cell differentiation and proliferation.

## 1. Introduction

In comparison with individual cell suspensions, three-dimensional (3D) cell aggregates provide key advantages for the creation of functional microtissues [1,2,3,4]. The cell aggregation process gives rise to multicellular assemblies with arbitrary shapes and numbers of cells that include different types of structures based on their complexity level: clusters, spheroids, multicellular spheroids, and organoids. Compared to suspensions of isolated cells, such 3D cell aggregates provide cellular interactions that better resume the real organization of native tissues [5,6,7,8,9]. Furthermore, the co-culture of different cell types in these aggregates (such as endothelial or immune cells) can facilitate ECM secretion, promote pre-vascularized networks and allow more accurate recapitulations of physiological tissular microenvironments [10,11]. This perspective has strongly motivated the development of methods to generate large-scale constructs by organizing multiple cell aggregates in controlled architectures to achieve large-scale functional constructs [12,13]. Intriguing approaches such as 3D bioprinting and micro-robotic placements through magnetic, acoustic, and pressure actuation methods have been proposed in this direction. To our mind, a broad analysis of the technical concerns and scientific opportunities behind these first attempts could be fruitful for the bioengineers targeting the generation of functional tissues.

**Figure 1:**
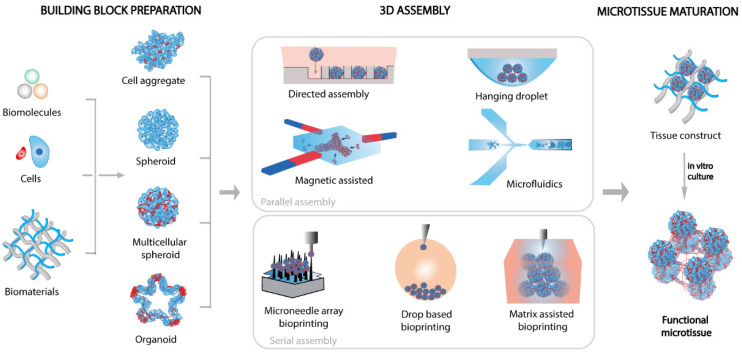
Illustration of microtissue fabrication by assembling in space different types of engineered cell aggregates. Emblematic assembly techniques are displayed with popular multicellular structures such as aggregates, spheroids, and organoids.

In this paper, we review the emerging spheroid/organoid handling technologies and their perspectives on building functional microtissues (Figure 1). We describe the recent and promising routes suggested in the literature and propose a synthesis of the challenges identified for regenerative medicine or drug assessment applications. We also address the improvements in the building of functional biological artifacts from cell aggregates compared to construction schemes based on individual cells. Finally, we discuss combinatorial methods for making pre-mature microtissue models of increased efficiency to shorten the healing time when implanted into humans.

## 2. Engineering Novel Building Blocks for Microtissue Formation

Despite considerable progress in tissue engineering, the development of relevant tissue models reproducing the cell population diversity, the cellular organization, and the environment found in organs is a major challenge. The behavior of mammalian cells in a tissue is indeed governed by specific three-dimensional (3D) microenvironments that involve a dynamic interplay between biochemical signals provided by the extracellular matrix (ECM), cell-cell interactions, and soluble factors. Moreover, the physical properties of the microenvironment, including stiffness, geometry, and topography act in concert with biochemical signals to impact cell fate, tissue homeostasis, and functionality [14,15]. Current 2D culture models have demonstrated the ability to form de novo extracellular matrices (ECMs) from seeded cells, but this planar configuration cannot reproduce certain key physiological features of the in vivo native environment, including the dynamic cell-ECM, cell-cell interactions, and the heterogeneity and complexity of cell architectures in 3D (e.g., tubular structures as intestine; hollow, nontubular, viscous organs as vagina; solid organs as liver and bone) [16,17]. To date, one of the major bottlenecks in tissue engineering is the organization in 3D of multiple cell types associated with specific matrix compositions to initiate the structural organization of tissues. In this respect, embedded mammalian cells within hydrogel materials have been largely investigated to generate 3D tissue structures [18]. This approach has attracted significant interest due to the tunability of the properties of the embedding material. This versatility has been used to mimic natural ECM to control the porosity of the microenvironment, which is crucial for cell proliferation and transplantation and to shape the biological artifact in 3D [19,20,21]. Many studies have attempted to engineer cell-laden structures with ideal geometric shapes that can induce tissue fusion to produce 3D constructs. There is now a deeper appreciation of the development of these small pieces of tissues, often called microtissues, to recreate the complexity of native architectures and favor their integration and maturation in vivo (Box 1). Even if they cannot fully mimic in vivo situations, microtissues built up from well-controlled and tunable matrices, and cell types have generated interesting results for organotypic studies and transplantation [11,22]. However, some limitations of this approach have been reported and discussed such as relatively low cell density, the lack of cell-cell interaction, and the poor diffusion of metabolites within the structure. Altogether these effects affect cell viability and impede the emergence of advanced tissular functions [23,24].

Box 1 **Definition of different types of cell aggregates.**

Cell aggregate: Cell aggregation is a generic term describing the clustering and adhesion of initially separate cells to form an aggregate. The aggregate morphology permits re-establishment of the cell-cell contacts normally present in tissues; therefore, cell function and survival are often enhanced in aggregate culture.

Spheroid: Spheroids are generated from primary cells or immortalized cell lines. The common composition is one cell type. However, multicellular spheroids loaded with different cell types can be formed when a culture medium is optimized for co-culturing.

Organoid: Organoids originate from tissue-derived adult stem cells, embryonic stem cells, or induced pluripotent stem cells, capable of self-renewal and multi/pluripotency. In suitable conditions, these immature cells are able to give rise to the different phenotypes present in tissues. Therefore, a key difference separating organoids from spheroids is that organoids better represent the in vivo cellular heterogeneity and physiological functionality of the organ.

Assembloid: Assembloids are the next generation of organoids. They combine organoids generated from different organs or different regions of an organ. Culture media optimization, mentioned for the generation of multicellular spheroids, is even more critical to assembling organoids.

The use of cell aggregates (Box 1) as a building block for the formation of microtissues requires the development of specific methods capable of creating 3D architectures while providing a suitable microenvironment to promote tissue maturation and reach a functional state. Methods such as directed assembly, magnetic assembly, microfluidic assembly, or hanging droplets benefit from spontaneous interaction mechanisms in confined geometries and provide parallel processes to assemble a large number of entities in a single step. These methods usually provide simple construction approaches, but they often lack the degree of accuracy and control required to create complex architectures with arbitrary arrangements. In this respect, bioprinting has recently emerged as a sequential but versatile technique for the creation of custom 3D configurations of cellular aggregates. Bioprinting gathers several methods that allow the direct placement of cells or cell aggregates with or without, together with a biomaterial mimicking extracellular matrix (ECM). It includes droplet-based or robotic handling methods that have shown considerable potential for creating complex architectures from cell aggregates.

Here, we outline several methodologies and summarize their merits and limitations for the creation of microtissue constructs (Table 1). These technologies will be further discussed in the following paragraphs.

### 2.1. Directed Assembly of Spheroids and Organoids

**Figure 2:**
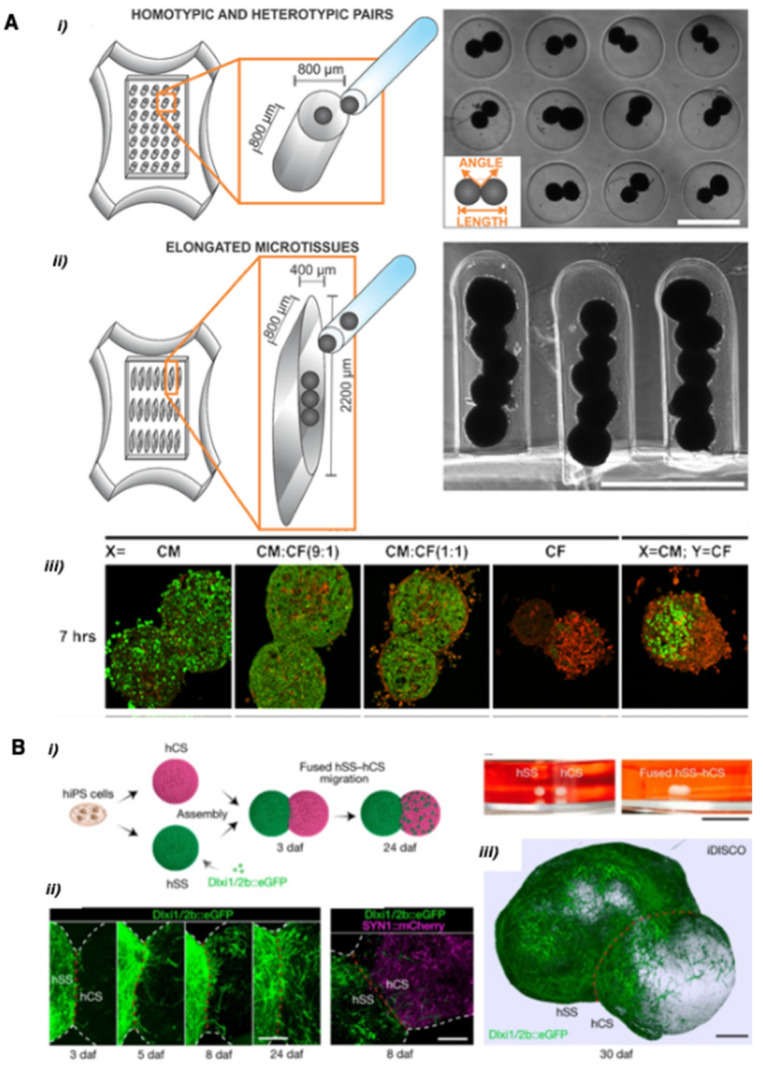
Directed assembly approaches. (**A**) Formation of cardiac spheroid pairs, and elongated microtissues. Cardiac fibroblasts (CF) and cardiomyocytes (CM) in suspension were co-seeded to the center of the hydrogel. (i) cylindrical microwells containing homotypic or heterotypic spheroid pairs (ii) elongated molds to form a larger building block. Scale bars: 800 μm. (iii) Cryosections of spheroids stained with antibodies recognizing α-sarcomeric actinin and vimentin for the CMs (green) and CFs (red), respectively. Scale bar: 50 μm. Reprinted from Ref. [25]. (**B**) Spheroid fusion of human cortical spheroids (hCS) and human subpallium spheroids (hSS). (i) Scheme of spheroid assembly and morphology of the spheroids before and after assembly. (ii) Time-lapse of migration from hSS into hCS (daf: days after fusion). (iii) 3D image of hybrid cerebral microtissue. Scale bar: 200 μm. Reprinted with permission from Ref. [26]. Copyright 2017, Springer Nature.

Self-assembly is usually defined as the autonomous organization of mobile objects into complex architectures [51,52]. Directed assembly involves the introduction of driving forces such as chemical bonding, physical interactions, geometrical confinement, or biological adhesion that can be tuned to orient the assembly into a desired structure or configuration [53]. This concept has been recognized as an efficient approach to ordering large numbers of mobile objects (e.g., microparticles, cells) in a parallel way and has been recently extended to spheroids. Kim et al. (2018) generated scaffold-free 3D cardiac microtissues composed of multicellular spheroids used as building blocks and arranged in different configurations such as homotypic or heterotypic pairs and elongated structures (Figure 2A). Using a construction strategy based on the confinement of spheroids within topographically patterned templates the authors demonstrated the construction of fused heterocellular cardiac tissue with interconnected morphologies [25]. Birey et al. (2017) applied the same method for the fusion of two forebrain organoids (Figure 2B) to mimic human brain development and demonstrate inter-neuronal migration [26].

Another report by Schuurman et al. (2016) investigated the placement of cartilage multicellular spheroids inside an amphiphilic poly(ethyleneglycol)-terephthalate/poly(butyleneterephthalate) (PEGT-PBT) scaffold. The spheroids were assembled to produce a large-scale cellular 3D construct [27]. The zonal cell distribution pattern was not preserved after 31 days of culture; however, the authors evidenced some lateral spheroid fusion resulting in abundant cartilaginous tissue formation [51].

This method provides considerable flexibility in using different multicellular spheroids and controlling their relative proportion [54,55]. This characteristic might be advantageous for rapid and parallel tissue assembly but one of the major obstacles is the lack of flexibility for the creation of arbitrary pattern geometries, in particular when targeting complex or sparse 3D architectures.

### 2.2. Magnetic Assisted Assembly

**Figure 3:**
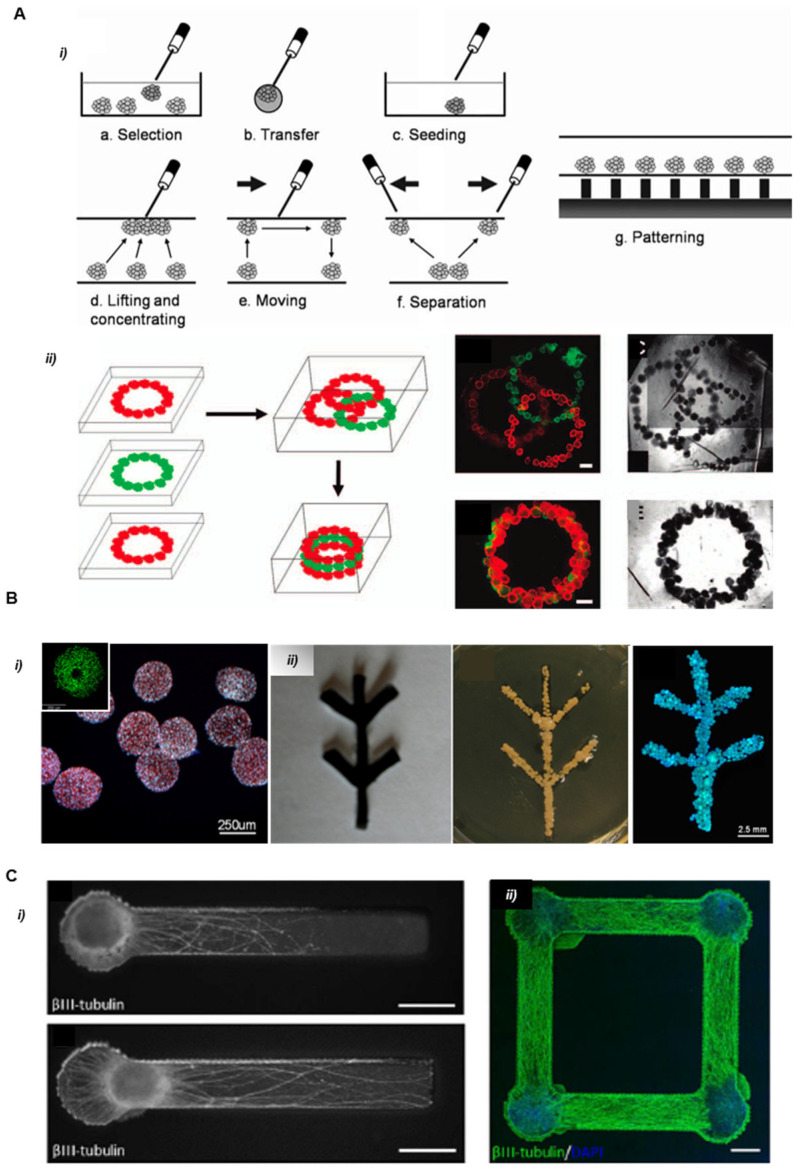
Manipulation of spheroids using a magnetic force. (**A**) (i) Schematic demonstration of spheroid manipulations that can be achieved by using a magnetic force. (ii) The scheme of layer-by-layer tissue reconstruction. Three pieces of human microvascular endothelial cell spheroids in Mebiol gel were stacked into an overlapped or aligned three-layered structure. Scale bars, 500 mm. Reprinted with permission from Ref. [29]. Copyright 2008, Mary Ann Liebert. (**B**) Patterning using paramagnetic particles (i) Size distribution and viability (inset) of superparamagnetic iron oxide nanoparticles (SPION) loaded endothelial cell spheroids. (ii) Light microscopy of magnetic template for the fusion of the spheroids (left panel). Magnetic assisted assembly of the spheroids at 48 h (middle panel). Confocal microscopy of preliminary fusion spheroids at Day 10 (right panel). Scale bar 2.5 mm. Reprinted with permission from Ref. [28]. Copyright 2013, John Wiley and Sons. (**C**) Magnetic assembly of central nervous system (CNS) spheroids. (i) Phase images of neural constructs (βIII-tubulin, white) indicate that the positioning of spheroids in constructs is more accurate with magnetic bioprinting (top panel) than manual placement with pipet alone (bottom panel). Scale bar 500 μm. (ii) Confocal imaging showing localized cell bodies (blue) and extending neurites (green) demonstrating the accurate positioning of multiple spheroids in the same construct using a multi-magnet tool. Scale bar 200 μm. Reprinted with permission from Ref. [30]. Copyright 2009, IOP Publishing.

Magnetic-driven positioning is a non-contact method to assemble spheroids. The inclusion of magnetic nano/microparticles is required in order to manipulate and position cell aggregates in levitation. The magnetic forces are involved to create specific attractive forces with templates of magnets that can be designed by computer-assisted methods (Figure 3). The organization can be obtained by labeling the cells with magnetic particles or by adding these particles to the cell culture medium [28,56,57]. The magnetic forces are used to maintain the formed 3D architecture while the fusion of the cellular building blocks occurs [29]. Magnetic assisted assembly can be easily upscaled, and combined with other methods such as hydrogel photopatterning [30], but it remains limited by the long-term presence of magnetic particles within the tissue. In a recent study conducted at the International Space Station, undesirable cytotoxic effects of the particles were reduced by using low-toxicity Gd^3+^ salts and performing the assembly in microgravity. The latter was successfully reported by Parfenov et al. (2020) in constructing 3D cartilage tissue incorporating human chondrocytes. The fusion of chondrospheres was observed in a non-toxic paramagnetic Gd^3+^ cell culture medium overcoming the gravitational force constraint [31]. This method is also useful for positioning spheroids on complex patterns with a possible stacking and 3D tissue generation [58,59].

### 2.3. Microfluidic Handling

**Figure 4:**
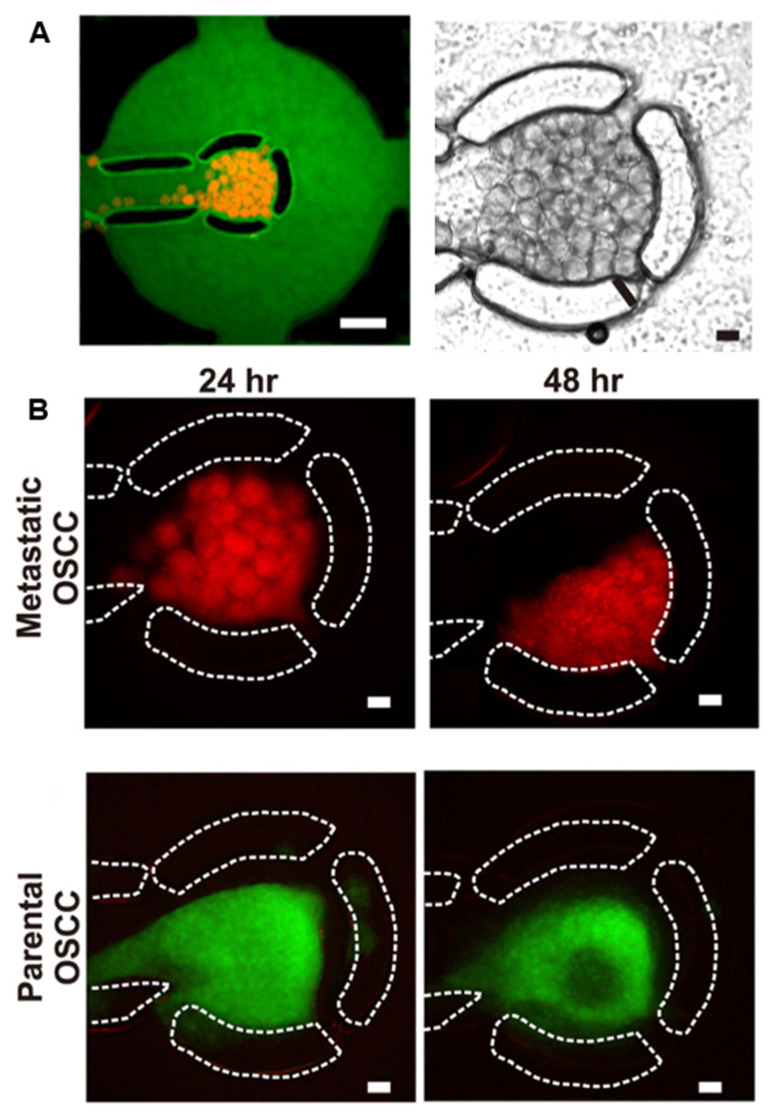
Visualization of metastatic oral squamous cell carcinoma (OSCC) spheroids immobilized within the cell culture chamber by (**A**) fluorescence (left) and light transmission (right) imaging. Culture medium was spiked with FITC-tagged BSA to visualize the cell culture chamber. Scale bar = 500 μm. (**B**) Fluorescent images of metastatic and parental HN137 OSCC spheroids in the microfluidic device after 24 h and 48 h of perfusion culture. Scale bar 100 μm. Reprinted with permission from Ref. [32]. Copyright 2009, IOP Publishing.

Microfluidics has emerged as a powerful tool for the controlled fabrication and programmed assembly of living building blocks [60]. In particular, the use of convective flow in confined geometries provides a simple and easily parallelized route toward the assembly of mobile objects in a suspension. This approach has first been applied to the construction of spheroids or organoids starting from individual cell suspensions. More recently this approach was extended to multi-spheroid constructs. In that respect, Ong et al. (2017) proposed a microfluidic cell culture device capable of directly immobilizing and maintaining the viability and functionality of 3D multicellular spheroids [32] (Figure 4). Patient-derived parental and metastatic oral squamous cell carcinoma OSCC tumor and human HepG2 hepatocyte spheroids were assembled and cultivated for up to 72 h with good viability and functionality. Whereas the evolution of the spheroid assemblies towards a microtissue could not be demonstrated, the metabolic activities of HepG2 spheroids cultured in the 3D printed device were significantly higher than those of static 2D, which were consistent with previous reports that 3D perfusion cultures enhance the liver-specific functions of hepatocytes [32]. These results also demonstrated the advantages of microfluidic culture systems for drug screening purposes or improving culture conditions toward tissue implantation and regeneration.

### 2.4. Programmable Assembly in Hanging Droplets

A straightforward approach to the formation of a spheroid is the hanging-drop method. Cells are suspended in droplets of the medium, where they develop into coherent 3D aggregates. In addition to being simple, the method eliminates surface interactions with an underlying substratum, requires only a few starting cells, and is highly scalable and reproducible. For the same reasons, this method has been applied to the co-cultivation of mixed cell populations and the fusion of cell aggregates.

In a recent article, Sloan et al. (2018) demonstrated the ability to generate region-specific spheroid models to study human brain development [33]. The authors have produced subdomain-specific forebrain spheroids from human pluripotent stem cells (hPSCs) and shown how to combine the neural spheroids (human cortical spheroids (hCSs) and human subpallial spheroids (hSSs)) in vitro to assemble forebrain assembloids that recapitulate the interactions of glutamatergic and GABAergic neurons seen in vivo and form physiologically relevant connections when assembled together. For that purpose, spheroids were fused by confinement in the bottom of a 1.5-mL Eppendorf tube, providing a simple and highly parallelized method for the creation of neural assembloids. Using a similar approach, Andersen et al. (2020) have demonstrated the assembly of derived organoids resembling the cerebral cortex or the hindbrain/spinal cord with human skeletal muscle spheroids to generate 3D cortico-motor assembloids [34]. The authors envision that his three-way system could develop a human cellular model of spinal cord injury (SCI) or be applied to assembloids of various parts of the central nervous system to bring insights into understanding its underlying developmental mechanisms into identifying therapeutic strategies.

**Figure 5:**
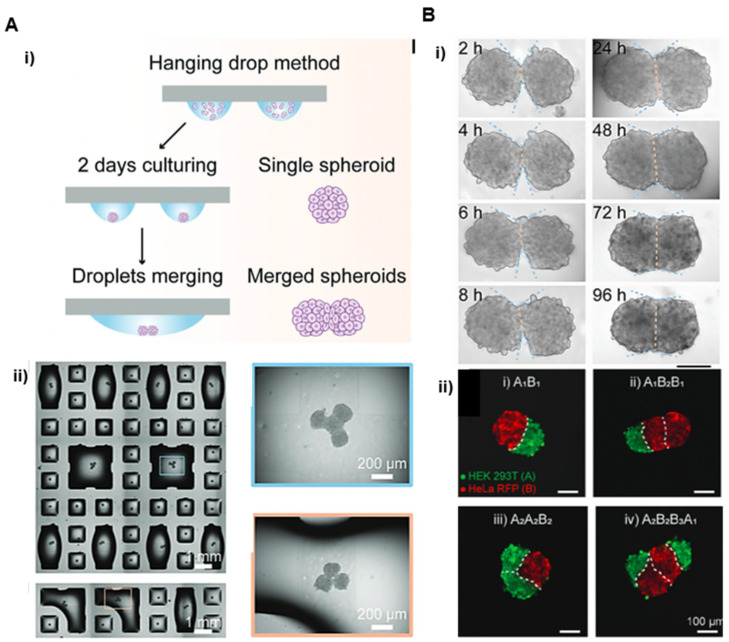
Programmable hanging drop method to form arrays of spheroids Reprinted with permission from Ref. [35]. Copyright 2020, John Wiley and Sons. (**A**) Merging of adjacent droplets. (i) Schematic of the hanging drop method to form arrays of cell spheroids using hydrophilic spots divided by superhydrophobic borders (ii) Micrograph of an array containing 2, 3, and 4 merged droplets (left side) and enlarged images of fused spheroids in merged droplets after 24 h (right side). (**B**) Examples of multi-spheroid architectures formed by this method. (i) Micrographs of two HepG2 spheroids fusing step by step over 96 h. (ii) Fluorescence microscopy images of hetero-spheroid architectures built from two different cell lines (HeLa cells expressing RFP and HEK 293T stained with green 24 h post merging. Scale bars 100 μm.

More recently, Cui et al. (2021) have developed a droplet microarray (DMA) platform that enables the parallel generation of cell spheroids using hanging drop methods [35]. From the conceptual point of view, this approach is similar to conventional strategies used for spheroid preparation that provide geometrical confinement in a cell suspension thanks to the shape of the drop interface. The authors advantageously combined this strategy with the planar arrays of hydrophilic patches that permit to generate, where the drops were developed and were used as anchoring structures separated by superhydrophobic barriers. Spheroids were generated by dispensing suspended cells in 50 to 300 nL liquid volumes and exploiting the gravity-driven aggregation process occurring at the droplet-air interface. By engineering specific DMA with controlled size and distances, it is then possible to add controlled volumes of the medium into neighboring droplets to selectively induce a spontaneous merging. Finally, the two initially separated spheroids were confined at the droplet base to promote their fusion. The combinatorial capacity of this method has allowed the creation of binary, ternary and quaternary assemblies (Figure 5). The method was applied to the study of the fusion of HepG2 spheroids or to the study of Wnt signaling propagation between 3D spheroids with varying cell compositions (HepG2, HeLa, and HEK 293T) first on double and further on triple spheroid complexes [35]. This method opens the way for high throughput and combinatorial investigations for rare and limited cell types such as primary patient-derived cells. It is an open droplet microarray platform that enables the structure of interest to be easily retrieved during experiments. However, it is poorly adapted to the creation of larger dimension multi-spheroid assemblies with controlled 3D architectures due to the formation of necrotic core induced by the limited diffusion of oxygen and nutrients.

### 2.5. Bioprinting

Bioprinting is an additive construction process used to deliver and spatially organize cells by stacking and assembling them into 3D architectures through different methods. This process is applicable to individual cells, cell aggregates, and spheroids with or without a supporting biomaterial. The process can be automated, thus allowing it to handle large quantities of biological materials and to create 3D arrangements with a resolution down to the cell level with high reproducibility [22,61,62].

Current conventional cell printing systems (extrusion, inkjet, or laser-assisted method) offer a resolution below 50 μm, but they imply laborious multi-material printing strategies and lack the possibility of handling large cellular constructs such as cell aggregates, organoids, or spheroids. The concept of printing tissue spheroids was first introduced in 2008 [63,64,65]. Forgacs et al. (2008) precisely positioned multicellular spheroids by bioprinting onto a layer of collagen, known as a biopaper [65]. Since then, many reports have been detailing new automated methodologies for spheroid printing based on drop dispensing or on dedicated microgrippers [13,66,67,68]. In bioprinting technologies, homogeneity in the size distribution of spheroids is a critical parameter to make them processable or dispensable through a bioprinter head to prevent damage or clogging. Therefore, standardization of the spheroid dimension is largely desirable for continuous dispensing.

#### 2.5.1. Drop Based Printing

**Figure 6:**
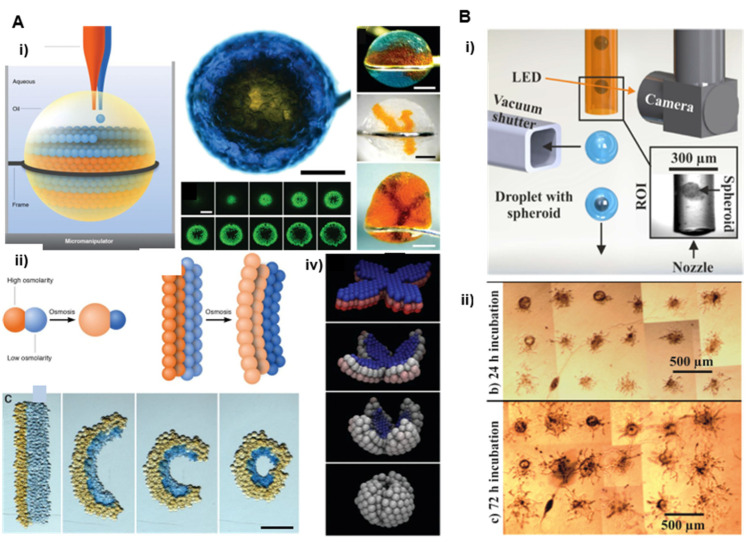
Droplet-based bioprinting. (**A**) Based on osmolarity gradients Reprinted with permission from Ref. [36]. Copyright 2013, The American Association for the Advancement of Science. (i) Droplet networks printed in bulk aqueous solution. The principle of aqueous droplets is dispensing into a drop of oil suspended in bulk aqueous solution (left side). Top view of a network printed in aqueous solution. A core of orange droplets is surrounded by a shell of blue droplets containing fluorescent pyranine (top middle). Confocal microscopy of the network in horizontal sections showing the fluorescent shell of droplets around the nonfluorescent core (bottom middle). Different types of networks printed in bulk aqueous solution (right side). Scale bars 400 μm. (ii) Principle of osmolarity gradients. (iii) Schematic of two droplets of different osmolarities joined by a lipid bilayer. The water flow through the bilayer causes the droplets to swell or shrink. (iv) Frames from a folding simulation of a network with a similar initial geometry Blue and red represent the lowest and highest initial osmolarities, respectively. White indicates the average of the two. (**B**) Single spheroid deposition setup Reprinted with permission from Ref. [37]. Copyright 2009, IOP Publishing: (i) Protruding transparent nozzle of a dispenser is primed with the spheroid solution. The generated droplet containing the spheroid can be dispensed onto a substrate if a spheroid is optically detected at the nozzle exit. If no single spheroid is detected, the droplet is aspirated by a vacuum shutter system. (ii) HUVEC spheroids were dispensed to defined positions and cultured for 24 h (top image) and 72 h (bottom image).

Droplet-based bioprinting uses methods similar to those involved in inkjet printers [68]. A single spheroid is loaded into a droplet of a bio-ink, which is used as a carrier for the printing and positioning of spheroids on surfaces (Figure 6). The key elements of drop-based bioprinting are the physical properties of the ink, the volume of the droplet, and the frequency of deposition, which are monitored by the dispensing head of the printer. Careful control has to be provided for the synchronization of the spheroid printing together with the movement of the printing nozzle. This method allows the fabrication of layers made of spheroids [12] together with more complex 3D structures (Figure 6A) [36]. More recently, Gutzweiler et al., (2017), adapted this scaffold-free bioprinting technique to automatically generate HUVEC spheroids via the hanging drop method [37]. The authors demonstrated a controlled deposition of single spheroids by drop-on-demand printing with interesting capabilities (1 μL droplet volumes and assembly of around 1500 HUVEC spheroids on a fibrin surface). The authors reported a spheroid printing efficiency of 97% (Figure 6B). The efforts devoted to the automation of the printing process have yielded a significant improvement in resolution, processing speed, and material saving that makes this technology upscalable. However, this method has limited performance in creating vertical or 3D complex structures and still does not provide cell densities mimicking native tissues.

#### 2.5.2. Bioprinting with Microneedle Arrays

**Figure 7:**
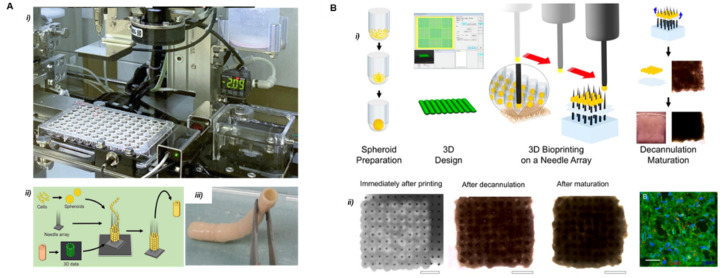
Principles of the Kenzan method. (**A**) Main components of the Kenzan bioprinting automated platform Reprinted with permission from Ref. [40]. Copyrihgt 2021, Springer Nature. (i) Robotic system. (ii) Illustration of methodology to create a scaffold-free cell-based vascular graft. (iii) A ready to implant cell-based vascular graft (diameter 5 mm × length 5 cm). (**B**) Bioprinting of cardiac patches using microneedle array principle Reprrinted from Ref. [46] (i) Schematic overview of biomaterial-free cardiac bioprinting process. (ii) Optical microscopy images at different steps and confocal microscopy of resulting cardiac patches. Scale bars 40 μm and 20 μm, respectively.

The use of removable scaffolds for the assembly of the multi-spheroid construct was first reported by Itoh et al. in 2015 [38]. This method is inspired by the Japanese traditional art Ikebana and uses arrays of needles as a temporary scaffold in which spheroids are immobilized by a robotic arm. This biomaterial-free method enables the accurate spatial organization of the spheroids or organoids in complex tissues analogs of practically any composition and organization [39,40,41]. The mechanical stability provided by the scaffold favors cell interaction, matrix secretion, and the formation of cohesive and functional tissue. The absence of biomaterials is also advantageous for implantation experiments, as it reduces the potential immune response. Itoh et al. used this method to produce models of the artificial aorta and implanted the resulting models in rats. The authors reported an endothelial network formation covering the inner surface of the tubular tissue after 5 days of implantation. Demonstrations of the printing and assembly of multicellular spheroids composed of human umbilical vein endothelial cells, human aortic smooth muscle cells, and human dermal fibroblasts were successfully achieved into scaffold-free small diameter tubular tissue (Figure 7A). This method provides a high accuracy thanks to the geometrical confinement permitted by the needle array. However, it imposes certain limitations regarding spheroid size to fit in the needle tip and gap between needles.

Moreover, needle-based bioprinting techniques are usually time-consuming and may not be adapted to build scalable constructs. The removal of the microneedle array is a critical step, especially if the mechanical cohesion of the cell is low. This difficulty results in low reproducibility and accuracy [38,41,42,43,44,45]

In 2017, Ong et al. demonstrated the bioprinting of spontaneously beating cardiac patches from multicellular cardiac spheroids using the microneedle array method (Figure 7B) [46]. The handling system was based on a bioprinting platform allowing the sequential manipulation of an individual spheroid by vacuum suction and delivery. It allows for the selection of spheroids with the desired dimension, for example, between 450 µm to 550 µm in diameter for micromanipulation, and rejects the use of all others that do not fit these criteria. This method provides more than 90% cell viability in a single-layered patch. Severe limitations were observed for the construction of multi-layer thick patches that reveal slow conduction velocity and a decrease in cell viability due to a lack of vascularization in the tissue. Moreover, the weak mechanical properties of the 3D bioprinted patches and their fragility during decannulation turned out to be a limitation for implantation applications.

It is interesting to notice that the methods presented here rely on the use of robotic handling to manipulate spheroids selectively and assemble them at precise locations. While most demonstrations rely on aspiration-based principles using pipetting platforms or bioprinters, this application has attracted a large interest in the field of robotic handling. Recent micromanipulation approaches have demonstrated their flexibility and accuracy for the handling of living objects such as cells, spheroids, and embryos while preserving their viability and integrity. In their recent work, Kozaki et al. (2020) developed a micromanipulator through high-resolution micro stereolithography that can capture and release a spheroid with minimal damage [69]. Spheroids were captured from the culture medium and trapped in a droplet of culture medium inside the cage-like microfingers. Injection of air through the fingers with controlled pressure was then involved in releasing the spheroid at the desired position, thanks to the elastic deformation of the fingers. This method allows the adaptation of the micromanipulator to match the dimensions and mechanical properties of the spheroid to be handled [69,70].

#### 2.5.3. Pressure Driven Bioprinting

**Figure 8:**
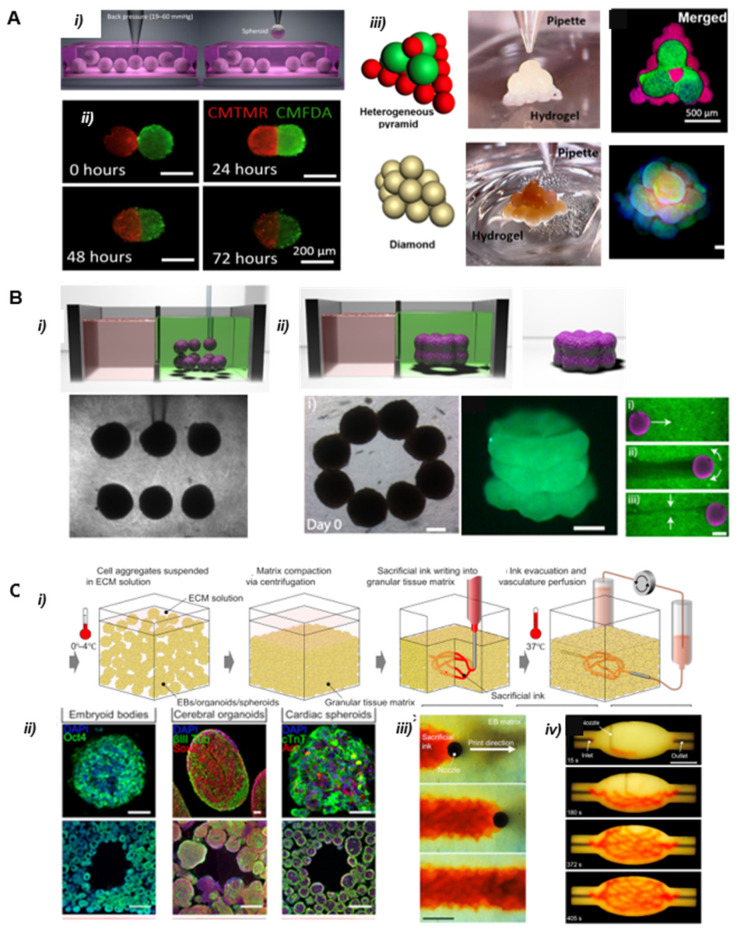
(**A**) Bioprinting of spheroids by a pressure-driven method Reprinted from Ref. [47]. (i) Illustration of the picking of individual spheroids by aspiration. (ii) Time-lapse images of the self-assembly process after bioprinting of 3T3 spheroids at different time points. (iii) Illustrations and micrographs of different shaped 3D printed structures with HUVEC and MSC spheroids represented in red and green, respectively. Dapi (blue) was used for staining of nucleus. (**B**) 3D bioprinting spheroids in supporting hydrogels Reprinted from Ref. [49]. (i) Spheroid deposition. (ii) Spheroid fusion in and removal of the structure from the gel after 4 days of culture. Scale bars 200 µm. (**C**) Sacrificial writing into a tissular matrix based on extrusion bioprinting Reprinted from Ref. [50]. (i) Illustration of the process. (ii) Examples of cellular construction for different OBB (organ building block) based matrices composed of embryoid bodies, cerebral organoids, and cardiac spheroids. (iii) Time-lapse of sacrificial ink (red) writing within a tissular matrix. (iv) Embedded 3D printing of a branched, hierarchical vascular network within a tissue matrix connected to inlet and outlet tubes, scale bar 10 mm.

Improving the physiological relevance of bioprinting and promoting vascularization and survival of the microtissue over long periods of time are key features in the perspective of tissue function and reimplantation. In this respect, a novel spheroid aspiration method was proposed and successfully tested on osteogenic spheroids. Ayan et al. (2020) developed an original assembly method using aspiration forces and high precision positioning onto hydrogel substrates [47,48]. The authors demonstrated vascular network formation by studying angiogenic sprouting of spheroids and osteogenic tissue engineering (Figure 8A). This method is compatible with a wide range of spheroid dimensions and is applicable to both scaffold-based and scaffold-free configurations. It provides a micrometric precision in spheroid placement in controlled architectures and is compatible with multi-spheroid construction. As previously mentioned, the biggest challenge of this kind of method is linked to the low speed imposed by the sequential handling of spheroids. Moreover, limitations arise for the assembly of complex 3D architecture that requires an external scaffold or sacrificial material to maintain the mechanical stability of the construct during printing. It is worth mentioning that the choice of supporting hydrogel (e.g., Coll-I or GelMA) around the spheroid is essential to promote adhesion and tissue integrity.

Another remarkable report by Daly et al. (2021) described a method using a supporting hydrogel to form high cell density microtissues by controlled spheroid fusion [49]. The properties of the supporting hydrogel enable precise positioning and holding of spheroids and high spheroid viability after printing (Figure 8B). The viscoelastic and non-adhesive nature of the hydrogel facilitates the fusion between adjacent spheroids into prescribed, stable structures. By mixing spheroids of different cell compositions, iPSC-derived cardiomyocytes, or primary human cardiac fibroblasts, the authors reported a model of focal cardiac fibrosis that replicates post-myocardial infarction pathologies with reduced contractile output and electrical synchronization.

The same kind of method has been implemented to manufacture thick tissues. Skylar-Scott et al. (2019) produced multicellular cardiac tissue (HUVEC cells and human iPSC-derived cardiac spheroids derived organoids) with engineered ECMs and embedded vasculature through bioprinting of a sacrificial bio-ink [50]. The developed method manipulates hundreds of thousands of organ building blocks (OBB) into tissular matrices with high cellular density (Figure 8C), in which hierarchical vascular channels can be introduced. Despite the need for large-scale production of organoids (~10^8^ cells/mL), this method enables the fabrication of perfusable organ-specific tissues of arbitrary volume and shape in a scalable manner.

**Figure 9:**
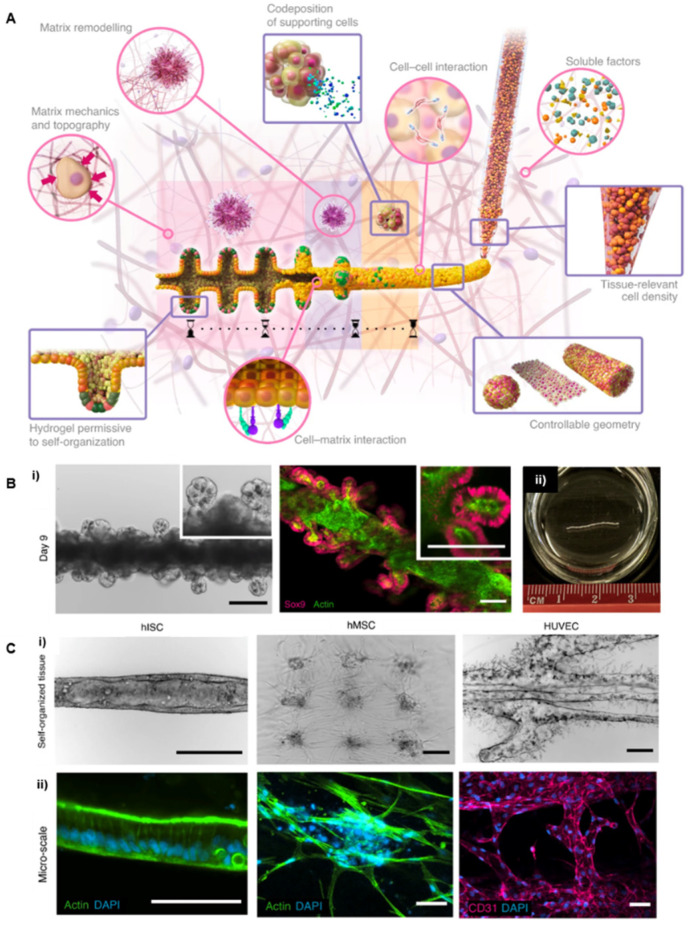
Bioprinting high cellular densities inside hydrogels. Reprinted with permission from Ref. [22]. Copyright 2020, Springer Nature. (**A**) Macroscopic intestinal printing principle. Multicellular self-organization was achieved directly inside the hydrogel environment. (**B**) (i) Bright-field and confocal microscopy of the intestinal tube showing stem cells forming the crypts on Day 9. Scale bars, 200 μm (left), 100 μm (right). (ii) Image of a centimetric intestinal tube. (**C**) Bright-field and confocal microscopy of the embedded patterns of hISC, hMSC, and HUVEC cells. (i) Bright-field images. Scale bars, 500 μm. (ii) Cells were labeled with DAPI (blue) and F-actin (green) or CD31 (pink). Scale bars, 250 μm (left) and 75 μm (right).

The bioprinting strategy also offers the possibility to print multiple cell types in the form of the concentrated cell suspension or cell aggregates obtained from dissociated organoids. Compared to spheroid printing, the reduced size of such aggregates prevents the risk of necrotic core formation and provides more flexibility for the cells to interact and evolve spontaneously towards a 3D architecture and functional tissue. Recently, Brassard et al. (2020) introduced a bioprinting concept adapted to the delivery of high density of stem cells or cell aggregates directly into extracellular matrices with a resolution down to the single line-level [22]. The authors showed the spontaneous evolution of printed intestinal stem cells (ISC) or mesenchymal stem cells (MSC) towards a tubular-shaped tissue reproducing the luminal morphology of the intestine. This extrusion-based system was also used for the organization of centimeter-scale tissues that comprise features such as lumens or branched vasculature. Morphogenesis could also be modulated by sequential deposition of the supporting epithelial cells and the organ boundaries of the gastrointestinal tract (Figure 9). Sachs et al. (2017) also used the fusion of intestinal organoids to generate centimeter-scale interconnected ‘mini guts.’ The authors generated macroscopic size intestinal tubes from small cystic organoids by embedding them in a floating collagen hydrogel to allow the spheroids containing stem cells to align and self-organize [71]. These two methods are inspiring examples of direct bioprinting in hydrogels allowing self-organization of the cell aggregates from millimeter to centimeter scales.

## 3. Conclusions and Future Perspectives

All the technological developments and related instruments presented in this review are highly encouraging. They suggest that manipulating and assembling building blocks such as spheroids or organoids is a possible route for generating relevant pieces of tissues that exhibit overall dimensions compatible with their use as advanced physiological models. Compared to the assembly of individual cell suspension at low concentrations, 3D cell aggregates provide cellular building blocks promoting cell-cell interactions, ECM secretion, cell differentiation, and tissue vascularization, thus leading to culture models that can better recapitulate the structure and function of native tissues.

This field of research activity is expanding, driven by the demand for the reduction of animal testing in agreement with the current guidelines and legislation recommending following the “3R” principles: “Replace, Reduce, and Refine” [72,73]. Fundamental studies of various biological mechanisms linked to specific functions of tissues, pharmacological drug testing, and toxicology, precision medicine or regenerative therapies represent examples of the broad spectrum of the possible end-users of this kind of technology. The current state of the art of this kind of method clearly shows that the field is in an exploratory phase mainly focused on the handling and patterning of cells and spheroids. Up to now, little attention has been paid to the collective behavior of cells and their adequate supply of oxygen and substrates according to the chosen technology, whereas the issue of necrotic core and the poor perfusion of the inner cell mass is clearly established [22,54,62]. Pending more complete experimental data, it is reasonable to speculate that the aggregation in macrostructure without a “technological” help to improve the providing in substrates will lead to this kind of limits in long term culture. At this level of the domain’s infancy, it is difficult to predict if a standardized method combining reproducibility, throughput, and versatility to all kinds of tissue constructs will emerge. We feel that according to the targeted tissue model (skin, brain, adipose tissue, bone, liver, intestine …), the topological constraints required to mimic the physiological situation will select different methods to adapt to the final target (in vitro bioassay or transplantation). The process of building cannot be distinguished from the final goal again. Indeed, the field is not limited by a lack of technological solutions: automation methods and microfabrication techniques provide a large reservoir of processes that can be implemented for building 3D multicellular building blocks while preserving cell viability. The main objective of the domain is rather to combine the engineering process together with the biological evolution of the cells inside the constructs that need to be favored as a functionality of the tissue. Successful methods will be those combining top-down (engineering) and bottom-up (biology) processes in such a way that the evolution of the living entities is guided efficiently towards a directed architecture exhibiting the key functional features of the physiological counterpart. In other words, the expectation from the technological process of assembling is not the generation of a final functional structure but rather the construction of a scenario of cell evolution in 3D where the engineered structure triggers the evolution of the multicellular spheroids leading to the emergence of a function. In such a vision, the time evolution of the produced construct is as important as its design, while careful attention has to be paid to the conditions of long-term culture to allow maintenance and self-organization of multiple cell types. Future progress in the field is therefore linked to the development of simulation tools capable of modeling the evolution of the spheroids and cell populations during long-term culture. The results of these simulations will be crucial for guiding the selection of the cells to be incorporated inside the manipulated spheroids and for guiding the design of the construct during the assembling process.

Future works for researchers in the field need to address some common objectives for all kinds of tissue constructs: (i) improve the capacities in the manipulation and organization of the spheroids in higher quantities and with a better spatial resolution. (ii) increase the efficiency (assembling or writing speeds) in terms of the ability to work with a substantial number of spheroids to mimic macro-scale tissues (iii) meet the multicellular need, which is mandatory in tissue engineering; and, finally, (iv) develop simulation tools for guiding the design of the architecture to reach functionality upon time self-evolution.

## Figures and Tables

**Table 1:** Comparison of assembly methodologies of cell aggregates for tissue constructs.

					Bioprinting		
Method	Directed Assembly	Magnetic Assisted	Microfluidic Handling	Hanging Droplets	Drop Based	Microneedle Arrays	Pressure Driven
**Assembly** **Process**	Parallel	Parallel	Parallel	Parallel	Serial	Serial	Serial
**Working principle**	Spheroids confinement in patterned templates or scaffolds	Inclusion of magnetic nano/ microparticles within spheroids	Use of convective flow in confined geometry to trap spheroids	Merging of neighboring droplets containing spheroids	Use of single spheroid loaded droplets as carriers	Use of a robotic arm to trap spheroids in an array of needles	Deposition of spheroids by aspiration system into, temporary or not, hydrogels
**Cell types**	Rat cardiomyocytes, rat cardiac fibroblasts, human pluripotent stem cells, equine cartilage cells	Microvascular endothelial cells, rat embryonic spinal cord cells	Patient-derived parental and metastatic OSCC tumor cells, Human HepG2 hepatocytes	Human HepG2 hepatocytes, HEK293T cells	Human microvascular endothelial cells (HMVEC), HUVECs	Human dermal fibroblasts, human aortic smooth muscle cells, micro-mini pig mesenchymal stem cells.	Human iPSC-derived cardiac cells, HUVECs, human mesenchymal stem cells, murine 3T3 cell line, murine intestinal cells.
**Culture duration post-assembly**	From a few hours to several weeks Up to 60 days with hiPSCs	From a few days to several weeks	Short term	Short term	Short term	Mostly a few days, up to two weeks	Up to three weeks
**Spheroid handling**	Contact	Non-contact	Non-contact	Non-contact	Non-contact	Contact	Contact
**Advantages**	Rapid tissue assembly Flexibility over the use of different spheroid types Controllable optical mapping Suitable for matrix rich tissues (bone, cartilage)	Compatible with hydrogel embedding Control of spheroid positioning inside complex patterns Possible stacking of multiple spheroids layers to generate 3D tissue	Fine control over microenvironment t 3D culture perfusion Compatible with drug screening purpose	Combinatorial approach Flexibility over the use of different spheroid types	High control of spheroid positioning Quality control for the generation of droplets loaded with individual spheroids Possibility to engineer self-folding droplets	High control of spheroid positioning Combinatorial approach Mechanical stability provided during tissue formation by the needles array	High control of spheroid positioning Microvasculature fabrication using sacrificial material Promotion of microtissue survival by scaffold-based approaches
**Limitations**	Low reproducibility Limited control over pattern geometry	Long-term presence of magnetic particles Potential cytotoxic effects	Poor control over 3D architecture Difficult to standardize and scale up for multicellular systems	Poor control over 3D architecture Low reproducibility Limited long-term maintenance with necrotic core formation	Non-physiological cell density Limited performance for complex 3D structuration	Low flexibility Not suitable for large aggregates Time-consuming Possible low cohesion of the bio-construct after micro-needle array removal low reproducibility	Low printing velocity due to sequential handling of spheroids Challenging maintenance of mechanical stability when associated with sacrificial material
**3D** **Positional accuracy**	Low	Medium	Low	Low	High	High	High
**Scalability**	Low	High	Difficult for multi-cellular system	Low	Medium	Low	Reduced by printing velocity
**Comments**		Ongoing developments to reduce particle cytotoxicity	Requires further developments for the evolution of spheroid assemblies towards forming a microtissue	Ongoing studies to increase the diffusion of oxygen and nutrients	Requires careful control over the synchronization of spheroid printing and the movement of the printing nozzle	Ongoing development of tailoring micromanipulators that can match spheroid dimensions	Requires careful attention of the choice of supporting hydrogel
**References**	[25,26,27]	[28,29,30,31]	[32]	[33,34,35]	[36,37]	[38,39,40,41,42,43,44,45,46]	[22,47,48,49,50]
